# Comparison of Antibiotic Resistance Profile and Biofilm Production of *Staphylococcus aureus* Isolates Derived from Human Specimens and Animal-Derived Samples

**DOI:** 10.3390/antibiotics8030097

**Published:** 2019-07-19

**Authors:** Maria Vitale, Paola Galluzzo, Patrizia Giuseppina Buffa, Eleonora Carlino, Orazio Spezia, Rosa Alduina

**Affiliations:** 1 Laboratorio Genetica dei Microorganismi, Istituto Zooprofilattico Sperimentale della Sicilia, 90129 Palermo, Italy; 2 Dipartimento Scienze e Tecnologie Biologiche, Chimiche e Farmaceutiche, Viale delle Scienze, University of Palermo, 90128 Palermo, Italy; 3 Laboratorio Analisi Baiata srl, via Capitano Francesco Sieli, 1, 91100 Trapani (TP), Italy

**Keywords:** *Staphylococcus aureus*, Staphylococcal toxins, mecA, antibiotic resistance, biofilm activity, MRSA

## Abstract

Background: The diffusion of antimicrobial resistance is a significant concern for public health worldwide. *Staphylococcus aureus* represents a paradigm microorganism for antibiotic resistance in that resistant strains appear within a decade after the introduction of new antibiotics. Methods: Fourteen *S. aureus* isolates from human specimens and twenty-one from samples of animal origin, were compared for their antimicrobial resistance and biofilm capability. In addition, they were characterized at the molecular level to detect the antimicrobial resistance *mecA* gene and genes related with enterotoxin, toxin, and biofilm production. Results: Both phenotypic and molecular analysis showed main differences among human- and animal-derived isolates. Among the human-derived isolates, more multidrug-resistant isolates were detected and *mecA* gene, enterotoxin, and toxin genes were more prevalent. Different genes involved in biofilm production were detected with *bap* present only in animal-derived isolates and *sasC* present in both isolates, however, with a higher prevalence in the human-derived isolates. Biofilm capability was higher in human-derived isolates mainly associated to the *sasC* gene. Conclusions: The overall results indicate that human *S. aureus* isolates are more virulent and resistant than the isolates of animal origin randomly selected with no infection anamnesis. This study confirms that selection for more virulent and resistant *S. aureus* strains is related to the clinical practice.

## 1. Introduction

*Staphylococcus aureus* is often found as a component of the human microbiota associated with skin, skin glands, and mucous membranes, particularly in the nose of healthy individuals [1,2]. In some cases, *S. aureus* causes a wide range of soft human infections [3], such as mild skin and soft tissue infections, as well as life-threatening pneumonia, bacteremia, osteomyelitis, endocarditis, sepsis, and toxic shock syndrome [4], and it is implicated in both community-acquired and nosocomial infections [2]. In addition to the infections listed above, *S. aureus* is often responsible for scalded skin syndrome and staphylococcal foodborne diseases [5,6]. 

In addition to causing infections in humans, *S. aureus* can also be the origin of infections in ruminants such as cattle, goats, and sheep, leading to clinical and subclinical mastitis. The pathogen spreads from the udder of the infected animal into raw milk, affecting the quality and quantity of milk and milk-derived products. Therefore, this pathogen represents a major economic problem for farmers and a serious problem for the dairy industry [7].

*S. aureus* pathogenicity depends upon its capability to produce and secrete different toxins and virulence factors that contribute to colonization and invasion of the host and bacterial spread [8]. The family of superantigen exotoxins is comprised of well-known secreted virulence factors, such as the staphylococcal enterotoxins (*se*), the toxic shock syndrome toxin 1 (*tsst-1*), and the exfoliative toxins (*eta* and *etb*). The latter are associated with staphylococcal scalded-skin syndrome [5]. Until now, more than 20 staphylococcal enterotoxins that can cause food poisoning or enterotoxin-like proteins have been identified [9].

In addition to the production of virulence factors, *S. aureus* genome shows enormous plasticity with the consequent acquisition of transmissible genetic elements, coding for resistance proteins. One example is the *mecA* gene that is present within the staphylococcal cassette chromosome *mec* (SCC*mec*) [2]. The *mecA* gene encodes an alternative penicillin binding protein, PBP2a [1], that makes the bacterial strain resistant both to methicillin (MRSA) and all other β-lactam antibiotics [2]. The ability to acquire horizontally resistant genes and the antibiotic pressure induces the emergence of multidrug-resistant (resistant to three or more classes of antibiotics) *S. aureus* strains, which are considered a significant concern for public health. 

Furthermore, it is well known that *S. aureus* produces biofilm, a thick extracellular exopolysaccharide layer which protects bacteria. Biofilm can be easily formed inside biomaterials as indwelling medical devices, often causing chronic diseases that are difficult to eradicate. Biofilm formation is a multifactorial event, controlled by quorum sensing and several proteins, such as the accessory gene regulator (Agr), the biofilm-associated protein (Bap), the intercellular adhesion protein (Ica), and the *S. aureus* surface protein (SasC).

More recently, antimicrobial resistance has been found even in previously unexplored environments, where antibiotic pressure is missing, and it has been demonstrated that food can serve as a vehicle for transmission of *S. aureus* to the human population [10,11]. Indeed, the comparison between human isolates and animal-derived isolates was performed also in other studies [12] suggesting that food, after handling and processing, could represent a source of human infection, and for food operators a source of food contamination.

The aim of this work was to compare the antibiotic resistance profile and biofilm production of *S. aureus* isolates derived from fourteen medical specimens and twenty-one animal-derived samples collected in Sicily. In addition to the phenotypic characterization, the isolates were compared for the presence of toxin genes and biofilm-related genes.

## 2. Results

### 2.1. Antimicrobial Susceptibility of S. aureus Human Isolates

Among the human isolates, twelve (85.7%) were resistant to benzylpenicillin, seven (50%) were resistant to erythromycin; six (42.8%) were resistant to clindamycin; five (36%) were resistant to oxacillin, cefaclor, ceftriaxone, ciprofloxacin and moxifloxacin; four (30.8%) were resistant to amoxicillin-clavulanic acid; three (21.4%) were resistant to gentamicin and tetracycline; and only one (7.1%) was resistant to sulfamethoxazole/trimethoprim (Table 1). Five human isolates (38.5%) resulted positive for cefoxitin screening, thus representing MRSA strains, and resistance to a larger number of antibiotics. There was no evidence of isolates that were resistant to linezolid, teicoplanin, vancomycin, tigecycline, and fusidic acid (Table 1). 

### 2.2. Antimicrobial Susceptibility of Animal-Derived S. aureus Isolates

Among the animal-derived isolates, eleven (52.4%) were resistant to benzylpenicillin, seven (33.3%) were resistant to tetracycline, three (14.3%) were resistant to ceftriaxone and gentamicin, and only one (4.7%) was resistant to lincomycin (Table 2). There was no evidence of isolates that were resistant to erythromycin, oxacillin, and vancomycin (Table 2).

Among the isolates, 7.1% of human- and 28.5% of animal-derived isolates were sensitive to all antibiotics used in this study. Regarding the human-derived isolates, 14.3% presented monoresistance, while 78.6% of the isolates showed a multi-antibiotic resistance. Among the animal-derived isolates, 4.8% showed a single resistance, 47.6% a double resistance, and 4.8% a multiple resistance. Three of the animal-derived isolates (14.3%) showed intermediate susceptibility to one or more molecules and were not resistant to any antibiotics (Table 3). 

**Figure 1:**
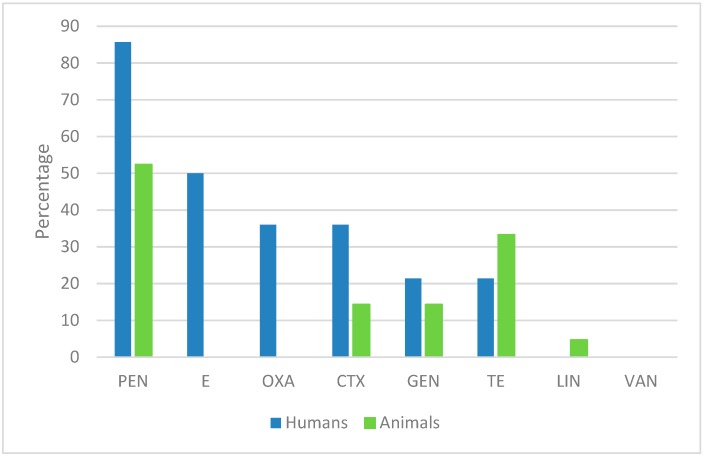
Percentage of human- and animal-derived isolates resistant to the antibiotics used in this study.

The comparison of the percentages of the antibiotic-resistant isolates showed that the prevalence of penicillin, erythromycin, oxacillin, and ceftriaxone is much higher in human isolates than in animal isolates. The opposite trend was registered for tetracycline resistance. No resistance to erythromycin, oxacillin, and vancomycin was found in animal-derived isolates, and no resistance to lincomycin and vancomycin was found in the human-derived isolates (Figure 1).

### 2.3. Biofilm Production

**Figure 2:**
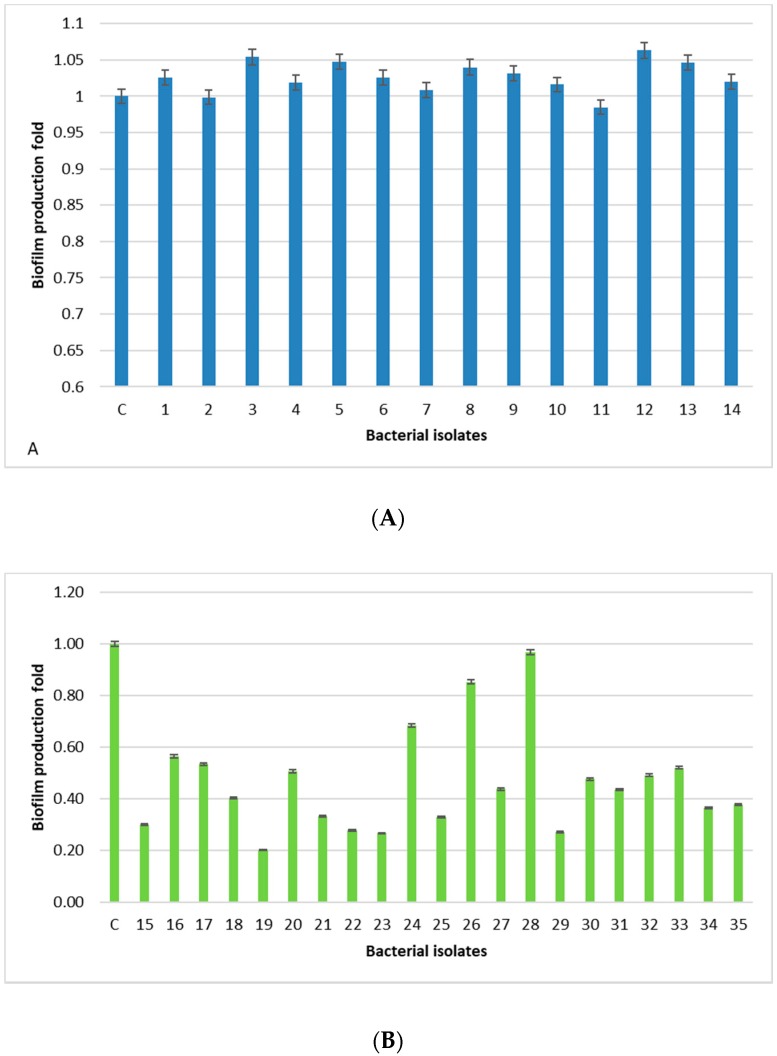
Biofilm production in human- (**A**) and animal-derived isolates (**B**). C represents the positive control. Standard deviations are indicated. Each experiment was performed in triplicate. Biofilm production was measured as the absorbance at 600 nm wavelength after crystal violet staining of the tubes containing the cultures.

All human-derived isolates (1–14) produced biofilm, as much or more than the positive control, represented by the *S. aureus* strain ATCC 25923, a strong biofilm producer (Figure 2A). In particular, isolates 3, 5, 12, and 13 produced the most biofilm. On the other hand, all the isolates derived from animal samples (15–35) were weak or moderate biofilm formers (Figure 2B). Isolate 28 produced as much biofilm as the positive control. The *S. aureus* human-derived isolates produced more biofilm than the animal-derived isolates, and there is a significant difference between the two groups (*p* < 0.0001).

### 2.4. Detection of Virulence and Biofilm Related Genes of S. aureus 

The presence of *mecA*, toxin (*sea-sej*, *tsst-1*, *eta* and *etb*), and four biofilm-related (*agr, bap, ica, sasC*) genes was investigated in all isolates by using PCRs. Five human-derived isolates (35.7%) positive to *mecA* gene PCR amplification were found. One human-derived isolate (7.1%) resulted positive for *tsst-1* gene and nine isolates (64.2%) resulted positive for genes encoding the enterotoxins. In particular, the *sei* and *seg* genes were frequently detected together (57.1%), followed by *seh* present in two isolates (14.2%), and by *sej*, *tsst-1,* and *see* detected only in one (7.1%). One human-derived isolate (7.1%) resulted positive for genes encoding the exfoliative toxins, *eta* and *etb*. The simultaneous presence of several virulence genes was found in ten isolates (71.4%) as reported in Table 4. The *agr* gene was found in one isolate (7.1%), *ica* in two isolates (14.2%), and *sasC* in eight isolates (57.1%). 

Regarding the animal-derived isolates, none of them resulted positive for *mecA* gene, while five isolates presented *tsst-1* gene (23.8%), six isolates (28.6%) resulted positive for genes encoding enterotoxins; specifically, three isolates (14.2%) presented *sea* gene, two isolates (9.5%) presented *see* gene and one isolate (4.8%) presented *sec* gene. Three isolates resulted positive for both *eta* and *etb* genes, two isolates (9.5%) presented only *etb* gene, and one isolate (4.8%) presented *eta* gene. Two isolates (9.5%) were positive through PCR amplification to *bap*, eight isolates to *sasC* (38%), and sixteen isolates to *ica* (76.2%). 

## 3. Discussion

In this study, we report phenotypic and molecular analysis carried out on human- and animal-derived *S. aureus* isolates collected in Sicily. Our results demonstrate that *S. aureus* isolates from human specimens were multi-resistant to antibiotics and produce more biofilm than the isolates collected from animal-derived samples. The high percentage (78.6%) of the human-derived isolates with multiple antibiotic resistance is in accordance with a recent study carried out in Serbia [12]. On the other hand, in other studies the animal-derived isolates showed a higher biofilm production than human-derived isolates [13]. The percentage of animal-derived isolates (47.6%) displaying multidrug-resistance was lower than those found in studies performed on other samples of animal origin. Indeed, in a study conducted on meat and dairy products collected in Puglia (Italy), 68.8% of the isolates were resistant to at least one antibiotic [14], and in another study performed on meat and poultry in the United States, 52% of the isolates were multi-resistant [15]. It is possible that the prevalence of an extensive and traditional farm management for ruminants in Sicily assures a lower circulation for multidrug-resistant clones, especially in healthy animals and in food derived from healthy animals, as in this study. In a previous study, in Ragusa Province in Sicily, on cows with mastitis reared in semi-intensive management, a higher prevalence of MRSA was detected [16]. 

In our previous study [17], resistance to penicillin was the most diffused in both human- and animal-derived isolates, even if a higher percentage (86%) was observed in human-derived isolates. 

Besides penicillin, human-derived isolates showed a high prevalence of resistance to erythromycin (50%), similar to results obtained on isolates collected from patients with early postoperative orthopedic implant-based infections [18] (erythromycin 82%) and from swine, farmers, and abattoir workers [19] (penicillin 96%, erythromycin 80.7%), even if the percentage of antibiotic resistant in our isolates was lower. In addition, we found less MRSA (35.7%) with respect to a study conducted on *S. aureus* isolates from skin and soft tissue infection, bloodstream infection, and lower respiratory tract infection collected from South Italy (40.7%) [20]. By comparing our results with this latter study, we found an increase of resistance to clindamycin (42.8 vs. 33%) oxacillin (36 vs. 0.9%), tetracycline (21.4 vs. 12.6%), and sulfamethoxazole-trimethoprim (7.1 vs. 3.2%) and a decrease of erythromycin (50 vs. 65%), moxifloxacin (11 vs. 72.3%), and gentamicin (21.4 vs. 39.5%) resistant isolates. A similar percentage of vancomycin, linezolid, and tigecycline resistant isolates was obtained.

With regards to animal-derived isolates, most strains showed resistance to penicillin (52%) and tetracycline (33%) in according with previous works [11,17,20,21,22,23]. By comparing our results with those obtained in a previous study [6] which reported the antimicrobial profile of 80 isolates collected between 1998–2014, we found an increase of resistance to penicillin (52% vs. 35.7%), tetracycline (33% vs. 20%), ceftriaxone (14% vs. 3.7%), and gentamicin (14% vs. 4%), which suggested the spread of resistance. In contrast, the percentage of lincomycin resistant isolates (4.7% vs. 3.7%) was unchanged and erythromycin resistance was lower in the new isolates (0 vs. 2.5%). We cannot rule out that the low number of isolates can be a bias for this result. Moreover, we found that 43% of the animal-derived isolates had an intermediate susceptibility to erythromycin, suggesting that this antibiotic should be used in a controlled manner even in veterinary practice.

All human-derived isolates are biofilm formers, with the isolates 3, 5, 12, and 13 being the strongest, whereas, isolates derived from food, dairy products, and animal tissue samples have a weak/moderate biofilm capability, except for one isolate (28) showing a level comparable to the positive control. The lowest prevalence of the *bap* PCR positive animal-derived samples (9.5%) is in accordance with the low ability of these isolates to produce biofilm, since *bap* could facilitate biofilm production in mastitis [24]. Moreover, the higher percentage of animal samples (76.2%) containing the *ica* locus, with respect to the 14.2% of human-derived isolates, is in accordance with a study carried out in Iran [13]. In this study, *sasC* gene is involved, but not essential, in the biofilm formation process in *S. aureus* in accordance with Schroeder [25]. In fact, we found this gene in eight human-derived isolates (57%) and eight isolates derived from food, dairy products, and animal tissue samples (38%). 

Antimicrobial spreading is a significant concern for public health worldwide. We analyzed the human- and animal-derived isolates for antibiotic resistance and virulence factors. The presence of the *mecA* gene was quite diffused (36%) in human-derived isolates and very low in animal-derived isolates (5%). 

Striking differences between human- and animal-derived isolates were also found in the relative presence of enterotoxin genes. Interestingly, in human-derived isolates the simultaneous presence of the enterotoxin genes, *seg* and *sei,* was quite frequent (50%). In our previous study, only a single animal-derived isolate and four human-derived isolates derived from a severe poisoning case showed the simultaneous presence of *seg* and *sei* [6]. In another recent study, *sei* was the second most diffuse enterotoxin in humans [12]. In addition, the animal strains showed the simultaneous presence of *sea* and *see* at 14.3%, according to another study on enterotoxin-producing *S. aureus* isolated from mastitic cows [26].

It is possible that the main reason for these differences is that human isolates are derived from symptomatic non-hospitalized patients subjected to microbiological controls while those derived from animals are part of a systematic screening. However, the results of this study confirm the importance of controlling antibiotic use in medical and veterinary practice. Although the *S. aureus* human-derived isolates could be more virulent for their antibiotic resistance, biofilm production, and presence of virulence genes, our results suggest monitoring the animal-derived isolates, since they are developing a greater resistance to the most commonly used antibiotics. 

## 4. Materials and Methods 

### 4.1. Clinical Sampling

Thirty-five *S. aureus* strains were isolated between December 2017 and February 2019 from different matrices, following standard laboratory protocols.

Fourteen *S. aureus* human-derived isolates and twenty-one isolated from animal-derived food were chosen for this analysis. (Table 5). Isolation was carried out by microbiological and biochemical methods [6]. Frozen cell glycerol stocks of *S. aureus* isolates were prepared as described in [27] and kept at −80 °C until use. 

### 4.2. Antimicrobial Susceptibility Tests of Bacterial Isolates

The susceptibility to antibiotics of the human-derived isolates was evaluated using the automatic VITEK^®^ 2 system (BioMérieux) following the manufacturer’s instructions. The microbial identification cards (ID) and the antimicrobial susceptibility tests (AST) were used. The susceptibility to 17 antibiotics (benzylpenicillin, amoxicillin-clavulanic acid, oxacillin, cefaclor, ceftriaxone, gentamicin, ciprofloxacin, moxifloxacin, erythromycin, clindamycin, linezolid, teicoplanin, vancomycin, tetracycline, tigecycline, fusidic acid and sulfamethoxazole-trimethoprim) was determined and interpreted according to the manufacturer’s instructions. Methicillin Rresistant *S. aureus* (MRSA) were determined by cefoxitin screening test. 

Regarding the animal-derived isolates, the antimicrobial susceptibility profiles for the main classes of antibiotics (penicillin, erythromycin, oxacillin, ceftriaxone, gentamicin tetracycline, lincomycin) were determined by using the Kirby–Bauer method using Mueller–Hinton agar (MHA) medium [6]. The results were interpreted in accordance with the standards for inhibition zone diameters for *Staphylococcus* spp. [28] using the clinical breakpoints compiled by EUCAST (http://www.eucast.org/clinical_breakpoints/). Vancomycin resistance was evaluated using the serial microdilution method.

### 4.3. Detection of SE (sea-see, seg-sei, sej, sep), tsst-1, eta, etb, mecA, and Biofilm Related Genes

Total DNA from *S. aureus* isolates was extracted by boiling the samples in 200 μl of TE buffer (10 mM Tris HCl, 1 mM EDTA, pH 8). The lysates obtained were numbered with an internal identification number. Three multiplex PCR assays were carried out to detect virulence genes in *S. aureus* [6,29]. PCR to detect *ica, bap,* and *sasC* genes were carried out following thermal profile and oligonucleotides reported in other studies [24,29,30]. Detection of *femA* was used as an internal positive control. Nucleotide sequences and PCR product sizes for the *S. aureus* gene-specific oligonucleotide primers used in this study are reported in Table 6.

### 4.4. Biofilm Formation Assay

The test for biofilm production was performed as described previously with some minor modifications [31,32]. In particular, after overnight growth of single colonies in 150 µL TSB at 37 °C in agitation for 24 h, the 96 microplates were washed twice with tap water. Then, 150 µL of a 0.1% of crystal violet in 0.9% w/v sodium chloride solution was added to each well and the plate was incubated for approximately 15 min at room temperature. After staining, the microplates were washed again two to three times to remove any trace of dye and then left to dry at room temperature for a few hours. Then, 150 µL of 33% acetic acid was used to solubilize biofilm forming cells and a microplate reader (GloMax^®^ multidetection system) was used to read the optical density at a wavelength of 600 nm. The average and standard deviation from a triplicate of each sample were determined. As a positive control, *S. aureus* ATCC 25923 was used. The SAS software (version 9.4) was used to calculate the *p* value. A *p* value ≤ 0.05 was considered as statistically significant.

## 5. Conclusions 

Our study demonstrated that *S. aureus* isolates from human specimens displays more resistance to antibiotics and produce more biofilm than the isolates collected from animal-derived samples. However, this study confirmed the importance of controlling antibiotic use in medical and veterinary practice and of monitoring the animal-derived isolates, since they are developing a greater resistance to the most commonly used antibiotics. 

## Figures and Tables

**Table 1:** Antimicrobial resistance profile of human isolates. The following antibiotics were tested by automatic VITEK^®^ 2 system: benzylpenicillin (PEN), erythromycin (E), clindamycin (CLIN), oxacillin (OXA), cefaclor (FAC), ceftriaxone (CTX), ciprofloxacin (CIP), moxifloxacin (MFA), amoxicillin-clavulanic acid (AMC), gentamicin (GEN), tetracycline (TE), sulfamethoxazole-trimethoprim (SXT), linezolid (LZD), vancomycin (VAN), tigecycline (TIG), and fusidic acid (AF). The susceptible (S), intermediate (I), and resistant (R) phenotypes are reported. P and N indicate the positivity or negativity to the cefoxitin screening. ND = not detected.

Isolate	Sample	PEN	E	CLIN	OXA	FAC	CTX	CIP	MFA	AMC	GEN	TE	SXT	LZD	VAN	TIG	AF	Cefoxitin Screening
1	Nail injury	R	S	S	S	S	S	S	S	S	S	S	S	S	S	S	S	N
2	Generic swab	R	R	R	S	S	S	R	R	S	R	S	S	S	S	S	S	N
3	Endoarticular liquid	R	R	R	R	R	R	R	R	R	S	S	S	S	S	S	S	P
4	Urine culture	R	S	S	S	S	S	R	R	S	S	S	S	S	S	S	S	N
5	Sore	R	R	R	R	R	R	R	R	R	R	S	R	S	S	S	S	P
6	Sputum	S	R	R	S	S	S	S	S	S	R	S	S	S	S	S	S	N
7	Sputum	R	S	S	S	S	S	R	R	S	S	S	S	S	S	S	S	N
8	Pharyngeal swab	R	R	S	S	S	S	S	S	S	S	R	S	S	S	S	S	N
9	Pharyngeal swab	R	S	S	R	R	R	S	S	R	S	S	I	S	S	S	S	P
10	Pharyngeal swab	R	ND	S	S	S	S	S	S	S	S	S	S	S	S	S	S	N
11	Pharyngeal swab	R	R	R	R	R	R	S	S	R	S	R	S	S	S	S	S	P
12	Pharyngeal swab	R	R	R	R	R	R	S	S	R	S	R	S	S	S	S	S	P
13	Pharyngeal swab	R	S	S	S	S	S	S	S	S	S	S	S	S	S	S	S	N
14	Pharyngeal swab	S	ND	ND	ND	ND	ND	ND	ND	S	ND	S	ND	ND	S	ND	ND	ND

**Table 2:** Antimicrobial resistance profile of animal-derived isolates. The following antibiotics were tested using the Kirby–Bauer method: penicillin (PEN), erythromycin (E), oxacillin (OXA), ceftriaxone (CTX), gentamicin (GEN), tetracycline (TE), lincomycin (L), and vancomycin (VAN). The susceptible (S), intermediate (I), and resistant (R) phenotypes are reported.

Isolate	Sample	PEN	E	OXA	CTX	GEN	TE	LIN	VAN
15	Cow milk	S	S	S	S	S	S	I	S
16	Cow milk	S	I	S	S	S	I	I	S
17	Cow milk	R	S	S	I	S	R	S	S
18	Goat milk	R	S	S	S	S	S	S	S
19	Goat milk	R	I	S	S	R	S	S	S
20	Sheep milk	S	I	S	S	S	S	S	S
21	Sheep milk	S	S	S	R	S	R	S	S
22	Sheep milk	R	I	S	I	S	R	S	S
23	Sheep milk	S	I	S	S	S	S	S	S
24	Sheep milk	R	I	S	R	I	I	I	S
25	Sheep milk	R	S	S	S	R	S	S	S
26	Sheep milk	R	S	S	I	S	R	S	S
27	Cheese	R	I	S	I	R	S	R	S
28	Cheese	R	S	S	S	S	R	S	S
29	Cheese	S	S	S	S	S	S	S	S
30	Cheese	R	I	S	I	I	R	I	S
31	Cheese	S	S	S	S	S	S	S	S
32	Cheese	S	S	S	S	S	S	S	S
33	Tuma (cheese)	R	I	S	S	S	R	S	S
34	Pecorino (cheese)	S	S	S	S	S	S	S	S
35	Food preparation	S	S	S	S	S	S	S	S

**Table 3:** Comparison of antibiotic resistance profiles of human and animal isolates.

	Number of Strains	Sensitive to All Antibiotics (%)	Single Resistance (%)	Double Resistance (%)	Multiple (≥3) Resistance (%)	Intermediate Sensitivity (%)
**Humans**	14	1 (7.1)	2 (14.3)	0 (0)	11 (78.6)	0 (0)
**Animals**	21	6 (28.5)	1 (4.8)	10 (47.6)	1 (4.8)	3 (14.3)

**Table 4:** Presence of the antibiotic resistance *mecA* gene, virulence (*sea-sep, tsst-1, eta,* and *etb*) and biofilm-related (*agr*, *bap*, *ica*, *sasC*) genes. ND indicates the analyzed genes were not detected with the used primers.

Internal ID	Sample	Virulence Genes			Biofilm-related Genes
		*sea, sec, see*	*seg-i, sej, sep*	*tsst-1, eta, etb, mecA*	*agr, bap, ica, sasC*
1	Nail injury	ND	*seg, sei*	ND	ND
2	Generic swab	ND	ND	ND	*sasC*
3	Endoarticular liquid	ND	*sei, seg, sej*	*mecA*	*ica*, *sasC*
4	Urine culture	ND	*seg, sei*	ND	*sasC*
5	Sore	ND	*seg, sei*	*mecA*	ND
6	Sputum	ND	ND	ND	*sasC*
7	Sputum	ND	*seg, sei*	ND	ND
8	Pharyngeal swab	ND	ND	ND	*sasC*
9	Pharyngeal swab	ND	*seg, sei*	*mecA*	ND
10	Pharyngeal swab	ND	ND	*tsst-1*	*agr, ica, sasC*
11	Pharyngeal swab	ND	*seh*	*mecA*	ND
12	Pharyngeal swab	ND	*seh*	*mecA*	*sasC*
13	Pharyngeal swab	*see*	*seg, sei*	ND	*sasC*
14	Pharyngeal swab	ND	*seg*, *sei*	*eta, etb*	ND
15	Cow milk	*see*	ND	ND	*ica*
16	Cow milk	ND	ND	ND	*ica*
17	Cow milk	*see*	ND	ND	*ica, sasC*
18	Goat milk	*sec*	ND	*tsst-1*	*ica*
19	Goat milk	ND	ND	*etb*	*ica, sasC*
20	Sheep milk	ND	ND	ND	*ica*
21	Sheep milk	ND	ND	ND	*ica*
22	Sheep milk	ND	ND	ND	ND
23	Sheep milk	ND	ND	ND	*bap*
24	Sheep milk	ND	ND	ND	*ica, sasC*
25	Sheep milk	ND	ND	*tsst-1*	ND
26	Sheep milk	ND	ND	ND	ND
27	Cheese	ND	ND	ND	ND
28	Cheese	*sea*	ND	*eta*	*ica, sasC*
29	Cheese	ND	ND	ND	*ica, sasC*
30	Cheese	*sea*	ND	ND	*ica, sasC*
31	Cheese	ND	ND	ND	*ica*
32	Cheese	ND	ND	*etb*	*ica, sasC*
33	Cheese	ND	ND	*tsst-1*	*ica*
34	Cheese	ND	ND	*tsst-1*	*ica*
35	Food preparation	*sea*	ND	*tsst-1*	*bap, ica, sasC*

**Table 5:** The 35 *S. aureus* samples collected from different specimens between December 2017 and February 2019.

Sample	n°
Cow milk	3
Cheese	8
Endoarticular liquid	1
Food preparation	1
Generic swab	1
Goat milk	2
Nail injury	1
Pharyngeal swab	7
Sheep milk	7
Sore	1
Sputum	2
Urine culture	1

**Table 6:** Nucleotide sequences of primers used in this study. The PCR product size is reported.

Gene	Primer	Oligonucleotide Sequence	Size of Amplified Product (bp)
*sea*	GSEAR-1	GGTTATCAATGTGCGGGTGG	102
	GSEAR-2	CGGCACTTTTTTCTCTTCGG	
*seb*	GSEBR-1	GTATGGTGGTGTAACTGAGC	164
	GSEBR-2	CCAAATAGTGACGAGTTAGG	
*sec*	GSECR-1	AGATGAAGTAGTTGATGTGTATGG	451
	GSECR-2	CACACTTTTAGAATCAACCG	
*sed*	GSEDR-1	CCAATAATAGGAGAAAATAAAAG	278
	GSEDR-2	ATTGGTATTTTTTTTCGTTC	
*see*	GSEER-1	AGGTTTTTTCACAGGTCATCC	209
	GSEER-2	CTTTTTTTTCTTCGGTCAATC	
*mecA*	GMECAR-1	ACTGCTATCCACCCTCAAAC	163
	GMECAR-2	CTGGTGAAGTTGTAATCTGG	
*eta*	GETAR-1	GCAGGTGTTGATTTAGCATT	93
	GETAR-2	AGATGTCCCTATTTTTGCTG	
*etb*	GETBR-1	ACAAGCAAAAGAATACAGCG	226
	GETBR-2	GTTTTTGGCTGCTTCTCTTG	
*tst*	GTSSTR-1	ACCCCTGTTCCCTTATCATC	326
	GTSSTR-2	TTTTCAGTATTTGTAACGCC	
*ica*	icaH-1m	TATACCTTTCTTCGATGTCG	700
	icaH-7c	CTTTCGTTATAACAGGCAAG	
*bap*	sasp-6m	CCCTATATCGAAGGTGTAGAATTGCAC	1000
	sasp-7c	GCTGTTGAAGTTAATACTGTACCTGC	
*sasC*	CHsasC1for	GCAACGAATCAAGCATTGG	600
	CHsasC1rev	TGACAGCACTTCGTTAGG	
*agr*	agrB1	TATGCTCCTGCAGCAACTAA	1070
	agrC2	CTTGCGCATTTCGTTGTTGA	
*femA*	GFEMAR-1	AAAAAAGCACATAACAAGCG	132
	GFEMAR-2	GATAAAGAAGAAACCAGCAG	
